# Hygienic Treatment of Patients

**Published:** 1868-03-01

**Authors:** J. Little

**Affiliations:** Le Roy, Ill.


### HYGIENIC TREATMENT OF PATIENTS.

BY J. LITTLE, M.D., LE ROY, ILL.

THERE is a vast number of important and interesting subjects in the domain of medical literature suitable for essays, but the selection of one was the most puzzling thing I had to doin discharging the duty assigned me by the worthy president of this honorable society. The principal reason for this was that every subject has been witten on by learned authors, and lectured upon by our medical teachers, so that any physician can turn to his library and open a book or journal, and read a more complete and able article than a country practitioner can write. It is for mutual improvement that we meet together, and I feel that it is the duty of each member to perform every task appointed him, and do all in his power to augment the interest and achieve the objects of our society.

I propose in this brief essay to say something about treating patients, as the treatment of every disease to which flesh is heir is fully discussed in our books, and I have not the vanity to believe that I have any thing new to offer on this topic, nor am I sure that what I shall say on that will be instructive or interesting. The treatment of diseases has been the great theme to which the attention of the medical savant as well as tyro, has been directed from the dawning of the science of medicine to the present time. To be cured of their maladies is what the afflicted want, and hence it is that all other subjects pertaining to medicine are considered subordinate to this, and the treatment of disease with drugs, medicines, charms, electricity, etc., has always been the great business of the "medicine man."

The treatment of patients is a theme not often discussed by medical societies; at all events, it has not been my good fortune to hear any such discussion. It may be said that the patient is always treated in treating his disease. That he should be treated is a self-evident proposition, it seems to me, but that he is properly treated in even following the directions laid down for the treatment of diseases by such authors as Wood, Watson, and Eberle, is evident to any person of ordinary intelligence. The symptoms, causes, clinical history, anatomical changes, diagnosis, prognosis and treatment are dwelt on at length, and maturely studied, and occasionally a few casual remarks are made in regard to cleanliness, dietetics and nursing the patient. Emetics, cathartics, bloodletting, stimulants, tonics, epispastics, opiates, etc., are spoken of minutely and the case dismissed. The whole discussion has been about the disease in its various aspects, and hardly a sentence has been uttered concerning the poor patient. His system is made the battle-field in which a war of extermination is waged against the disease by the doctor. Sometimes the struggle is fierce, obstinate, and bloody, and at other times it is exceedingly protracted, giving the combatants a splendid opportunity to display their tactics. To-day the physician seems to be master of the situation, and being encouraged he redoubles his efforts to drive his enemy to the wall, but on the morrow, when he revisits his patient, he finds that the disease has rallied its broken and scattered forces, and is making rapid and fearful advances. The gallant and faithful physician, believing his policy is right, determines to fight it out on "this line," and actively plies his pills, powders, tinctures, syrups, decoctions and teas, but still to his great astonishment the disease does not yield, but maintains its position with remarkable stubbornness.

The battle is now raging furiously between the doctor and the disease, each firmly fixed in his purpose, the latter to destroy the patient, the former to destroy the disease. Every organ in the body of the now prostrated patient bows submissively to the tremendous efforts of the physician to overcome his inveterate opponent. Finally, to make quick work of it, the doctor turns his heaviest pieces upon the strongholds of the disease, and accordingly fifteen grains each of *Calomel* and *Jalap* are sent rumbling along the alimentary canal to drive it out, a huge blister is applied over the spinal column or chest to draw it out, powerful diuretics or diaphoretics are administered to wash it out through the urinary apparatus or the pores of the skin, a pint or two of blood is withdrawn from the arm in hopes to capture it alive; and, while this bombardment is going on, the devoted doctor keeps his line of skirmishers well advanced, and maintains a continuous fire of small arms in the form of *Dover's powders*, *Sweet spirits of nitre*, *Paregoric*, *Rhubarb*, and tinctures and infusions of various kinds. And it sometimes happens that after the vigilant and untiring physician has completely exhausted his armamentarium, has fired the last round in the locker without any apparent effect, so far as curing the disease is concerned, and has retired from the scene of action in disgust, the disease sounds the retreat and quietly takes its departure, leaving the patient to himself—and he gets well. The patient is amazed at his recovery; his friends are delighted, and the doctor is nonplussed, and blames himself because he abandoned the patient so soon. Occasionally, while the physician is deeply absorbed in fighting the disease, the patient succumbs, leaving his friends in doubt which killed him—the doctor or the disease.

Now what I charge against the science of medicine, as generally taught and practiced, is, that disease is treated to the injury and neglect of the patient; but this is not true to the same extent of late years as in former times. I do not mean that the profession has directed too much attention to the study of disease, but too little to the care, comfort, and happiness of the patient. In my humble opinion, the sick person should receive the first consideration of the physician, and the disorder should be studied and treated afterward.

Suppose a person has been sick a week or two, and has lain in bed, with only such care as one unacquainted with the science and art of medicine would bestow. A physician is called to see him, and stops just long enough to make out a diagnosis and prescribe the usual remedies for the disease. Every day the visit is repeated, and medicines of various kinds are left for the treatment and cure of the malady, to be taken in obedience to very strict directions. The case lingers, and the patient and his friends become discouraged; the attending physician is dismissed, and another one called. When the second one comes, he takes a general survey and a comprehensive view of every thing around and in the house; he scrutinizes the bedding and clothing of the patient, the room, and all its furniture; he inquires into the kind of food and drink taken; he observes the cleanliness of every thing, and the light and ventilation of the sick chamber; he asks about the company and attendants; in short, he carefully examines into every thing that has any bearing in the case, and then directs that every thing hurtful be put away, and that the patient be placed in the most comfortable and sanitary condition possible. Then, unless the case is urgent, or there is a clear and positive indication for medicines, he leaves his patient without any in order to see what effect the changes he directed to be made will have by his next visit, when he will be more competent to decide what medicines to prescribe, if he should then think any are needed.

The scientific and conscientious physician makes haste slowly in treating the disease, but rapidly in treating his patient. He knows that the majority of sick people would recover just as quickly and surely without medicines as with them, if they were properly nursed and cared for. The charlatan teaches the people that they would surely die when sick unless they took medicines, and that all recoveries are attributable to them. The unprincipled fellow is always loudly boasting in public places of his ability to cure all diseases with his drugs and medicines. His heart is full of deceit and dishonesty; he has more brass in his face than brains in his head, and there is nothing too mean and contemptible for him to do in his trade of humbugging the people. It is among the ignorant in community that he has patrons, and what is needed to drive him and the vast herd of quacks and impostors that infest the land to honorable callings, is a general diffusion of knowledge. The regularly educated physician makes no pretensions, but modestly performs his professional duties, spending his leisure hours in study instead of gassing.

What is disease? Is it an entity or a condition? Does it invade the system of an individual as an enemy invades a country? Does it make its advances and attacks upon the organs of the body as an army does upon the forts, and cities, and territories of a nation? And must the physician proceed forthwith to fight the disease and expel it from the animal economy with powerful and hurtful agents, as the forces of a nation would drive an invading army from its possessions? Does the disease mean death and destruction to the patient, unless arrested in its career, or is it not rather a remedial effort on the part of the system to get rid of a morbid agent or process? Then if disease is not an independent existence, bent on the destruction and death of the patient, but a morbid state of the anatomy and physiology constituting the standard of health, are we justified in treating it by introducing into the system of the patient agents that are poisonous, and always more or less injurious to the organism, to the neglect of other means of acknowledged value and innocence under all circumstances? Is not the heroic plan of treating diseases opposed to the present teachings of physiology and pathology, as well as all the natural sciences? It is now the teaching of our medical schools that many of the most common diseases are self-limited, and tend to recovery from the first if not complicated with drugs, and the day may come when it will be discovered that all diseases are self-limited and tend to a favorable termination. No physician will deny, I presume, that hygienic management of patients upon all occasions is safe, proper, and necessary. Then is it not always better to travel a sure road, though it may not be the shortest one, to a given point, than to attempt to find a nearer one by wading through muddy water of unknown depth, and run the risk of losing your life?

We are taught that there is in every organized being a certain conservative power which opposes the operation of noxious agents, and labors to expel them when introduced into the system. This power has long been recognized; it is the "vis medicatrix naturæ" of Galen, and we all court its favor. Were it not for this power, sickness and death would be far more common than they are; nay, they would claim undisputed authority over every creature at the very threshold of animal life. Thus the natural tendency of organized bodies is to health and life, and not to sickness and death. Occasionally the causes of disease are too numerous and powerful for the conservative power or resistive force to repel, and sickness is the result. Or, in other words, the individual has violated the laws of his being to such an extent that an unnatural or forced state of his organism takes place, which we call disease. "The laws of nature are fixed and poison kills."

From the moment a female conceives, to the time when the fruit of that conception shall die of old age, it is under the dominion of natural laws. All things that exist came into existence in a regular and lawful way, and they live and die in obedience to law. Man's body is governed by the laws of nature, like every thing else, and he can not violate these laws without suffering the penalty. A man may break a law of society, or a civic law, and hide the fact; or, if the fact is known, the doer may be unknown, or if he is detected he may escape conviction; but nature's laws carry their punishments with them, and the soul that sins must die. Men live under the great material laws of the globe in which we dwell, and there is not a material law that a man can break and not suffer. If a man eats and drinks to excess, if he keeps late hours and revels in fashionable indulgences that fill him with all manner of buzzing pains, he sins against his own body, and suffers the pains and penalties of his own transgressions. Men are almost without law in respect to air, food, over-taxation, and neglect of every kind. They are perpetually committing transgressions which are in time the mother of other transgressions. Many social infelicities, such as irritableness, an unhappy temper, moroseness, are frequently the concomitants of simple indigestion or overwork, or an unduly excited state of the nervous system. Thus men begin by violating the laws of God in the body, and this extends to the violations of the laws of God which regulate the disposition, and these involve a great number of moral traits, and the ramifications are infinite. If a man rides or sleeps in a car or a chamber that is unventilated, or lives in a damp dwelling, and if pains and aches overtake him, if vertigo seizes him, if his brain is poisoned, or if inflammations and rheumatisms ensue, it is in vain for him to go and implead nature, "I did not know I was doing wrong, and I will not do it again." Neither his protestation of ignorance nor his promises of obedience will do any good or make a particle of difference. A man that breaks nature's laws must take the penalty. If a man swallows a dose of poison, it makes no difference that he can prove that he did not know that it was poison, it will destroy his life just the same.

The laws of nature are as immutable, beneficent, and ubiquitous as God their author, and every thing in the universe is governed by them. Every thing in existence has its function to perform, its destiny to fullfill, and as long as it obeys the laws of its being it will be well with it, and when it disobeys these laws it will suffer the penalty. I will then lay it down as a truism, that if an organized body obeys the laws of its being in every particular, it will run its course free from any punishments. No man is so foolish and wicked as to believe that the painful occurrences of this world come by chance, or are inflicted by the Great First Cause for amusement, or to appease his wrath. Then what we call disease comes as a penalty of violated laws, and is a legitimate production or sequence. All diseases are preventable if taken in time; and when a person is sick, he, or his parents, or his neighbors, has violated the laws of nature. Many a man goes on trampling on the laws of his body, and because the penalty is deferred, he thinks he is innocent, but by-and-by the punishments come down upon him. The punishments for the violation of natural laws can never be evaded, for they are self-executing punishments.

Believing then that diseases are the penalties of violated laws, which have we to deal with—the patient or the disease? Which is the active, responsible, intelligent object? If a man contract typhoid fever, variola, or pneumonia, he is to blame, not the disease. This comes as the consequence of trespassing against nature. The man attacks the disorder instead of it attacking him.

We hear a great deal said about diseases attacking people, as if they were rational, ferocious beings, existing some where till an opportunity occurred for them to make an attack upon some unlucky person who happened to go that way. The people have learned this language from the doctors, I presume; and you will hear them tell that a certain boy or girl, or some other individual in the neighborhood, was attacked by the measles, scarlet fever, or ague, and almost killed. The popular notion being that diseases are independent existences, going about the country in a similar manner and for a similar purpose as that individual spoken of in the Scriptures; as soon therefore, as an unfortunate person is attacked, a messenger is dispatched in great haste for a doctor, or some patent medicine, and an attack is at once made upon the ferocious disease, and immediately a fight ensues, which occasionally results not unlike the fight between the Kilkenny cats.

The physician's business is to cure the patient, or in other words, to take care of him till nature heals him. "Doctors cure but nature heals," is a maxim worth remembering always. How is the physician to cure the patient? Having a thorough knowledge of anatomy, physiology, chemistry, pathology and hygiene, and in fact of all the natural sciences, and comprehending the forces and processes of nature, and taking an understanding view of all the circumstances surrounding the patient, he will not be at a loss to know what is needed to effect his cure. The true physician has ever open before him the great volume of nature, and is continually conning its lovely and sublime lessons.

What law of his being has the patient violated to cause his sickness, is a question that suggests itself at once; for I hold that disease does not come by chance, nor is it self-imposed, but comes as a consequence of wrong-doing on the part of men. Not only must we inquire into man's relation to natural law, but his relation to the outside world, and to every thing affecting him. His age, occupation, nativity, parentage, temperament, food, clothing, condition, and in short, his past and present history in detail, must be known, and then a thorough rational and physical examination should be gone through with in order to find the nature, seat, and cause of the malady. This being carefully and methodically done, the treatment of the case will naturally follow.

It is a law in physics, that the cause being removed, the effect will cease; and this law holds good in a measure in the organic world. Then the first thing is to find the cause of the trouble and remove it, or remove the patient from under its influence. The relation which they sustain to each other must be broken up. There is a law of growth, development, maturity, decay and death in the organic world, a beginning, a career, and an ending, and judging from analogy we would conclude that all the morbid processes we call disease are self-limited; and observation and experience prove this. From what has been said, the inference would naturally follow that the cause of the disease being separated from the patient, if too great injury had not been inflicted, and he placed in the most favorable condition as regards the laws of nature, he would recover.

Understanding man's relation to all things that surround him, to the air he breathes, the water he drinks, the food he eats, the clothes he wears, the light and sunshine of day, the darkness of night, to activity and rest, wakefulness and sleep, and the proper exercise of the faculties of his mind, the thoughtful physician will at once see the importance of putting his patient in harmony with nature's forces, agents, processes and laws. All things were created by the Supreme Architect before man was made, and they were created for man because he was given dominion over them, and there is a perfect law of harmony and adaptation between man and every thing in his dominion. It was never intended that man should be a delicate, pale, sickly creature, but hale, strong, prosperous, and happy. It was never intended that two-thirds of the human family should die before living out one-third of man's allotted years. A sick man is a nuisance, but a healthy one is a monument of symmetry and power. It is by going to nature that we get just and right impressions and ideas in regard to medicine, law, and every thing else. When people live in the enjoyment of all the gifts of nature in their purity, there health prevails; but when these gifts become contaminated, as in camps, cities, and on ships, there disease and death prevail.

When we look around us and see how people live, see what they eat and drink, and wear, see what kind of houses they live in, and how little they know of themselves and all the things around them, do we wonder that they are sick? The only wonder is that they are not more sickly, and that the knell is not oftener heard. When people study themselves more and learn to obey the inexorable laws of nature, then will sickness decrease, and death will come at the end of a natural life in a natural way. Then is it not the duty of the physician to do all in his power to have anatomy, physiology, hygiene, and all the natural sciences studied as much as possible by the masses of the people. Prevention is a thousand times better than cure, is a truth which all should know, and believe, and practice.

(*To be continued.*)

